# Supplementary material for: Indoor Air Radon Concentration in Premises of Public Companies and Workplaces in Latvia
Source: Int J Environ Res Public Health. 2022 Feb 10;19(4):1993. doi: 10.3390/ijerph19041993 (PMC8871745; doi:10.3390/ijerph19041993)
Supplement: Supplementary file 1 [file ijerph-19-01993-s001.zip › ijerph-1551196-supplementary.pdf]

Table S1. Radon levels by municipality in Latvia (Bq/m³).

Nr.	Municipality	*n*	Mean	SD	Median	Q1	Q3	Min	Max
1	Aglona Municipality	3	150.33	70.49	167	73	-	73	211
2	Aizkraukle Municipality	1	27.00	-	27	27	27	27	27
3	Aizpute Municipality	4	41.00	18.92	42	23	59	21	60
4	Akniste Municipality	7	101.14	37.04	91	68	146	60	148
5	Aloja Municipality	11	166.82	82.04	193	91	238	33	273
6	Alsunga Municipality	4	61.50	20.82	61	43	80	43	81
7	Aluksne Municipality	9	48.44	12.82	48	37	62	31	66
8	Amata Municipality	N/A	N/A	N/A	N/A	N/A	N/A	N/A	N/A
9	Ape Municipality	10	56.90	24.79	51	42	73	18	107
10	Auce Municipality	5	113.20	52.08	89	69	170	66	170
11	Adazi Municipality	10	53.00	29.58	44	27	77	18	101
12	Babite Municipality	5	30.60	17.44	30	17	45	14	59
13	Baldone Municipality	5	91.80	74.51	79	36	155	16	214
14	Baltinava Municipality	4	77.00	7.44	79	69	83	67	84
15	Balvi Municipality	4	70.25	10.81	73	59	79	55	80
16	Bauska Municipality	8	95.13	50.33	80	49	148	43	174
17	Beverina Municipality	8	54.00	28.76	49	30	67	28	115
18	Broceni Municipality	4	6.75	6.29	5	3	13	2	16
19	Burtnieki Municipality	9	44.78	40.53	22	17	65	10	137
20	Carnikava Municipality	4	60.75	20.16	61	42	80	36	85
21	Cesvaine Municipality	10	47.30	16.40	46	32	58	27	80
22	Cesis Municipality	4	112.25	27.78	119	83	135	76	135
23	Cibla Municipality	4	120.00	116.52	82	36	243	28	288
24	Dagda Municipality	3	51.00	29.31	41	28	-	28	84
25	Daugavpils	30	66.23	69.73	41	25	92	14	359
26	Daugavpils Municipality	8	59.25	38.42	44	30	100	25	124
27	Dobele Municipality	9	66.89	42.65	69	23	108	19	128
28	Dundaga Municipality	N/A	N/A	N/A	N/A	N/A	N/A	N/A	N/A
29	Durbe Municipality	N/A	N/A	N/A	N/A	N/A	N/A	N/A	N/A
30	Engure Municipality	1	62.00	-	62	62	62	62	62
31	Ergli Municipality	4	106.25	56.58	96	58	165	50	183
32	Garkalne Municipality	5	30.60	9.45	27	24	40	23	46
33	Grobina Municipality	8	76.38	40.90	66	51	80	43	172
34	Gulbene Municipality	5	89.60	45.58	113	41	127	36	138
35	Iecava Municipality	10	40.00	16.22	41	28	54	6	58
36	Ikskile Municipality	N/A	N/A	N/A	N/A	N/A	N/A	N/A	N/A
37	Ilukste Municipality	10	108.10	116.92	54	41	171	30	393
38	Incukalns Municipality	10	68.10	29.22	67	51	83	26	125
39	Jaunjelgava Municipality	8	139.25	97.93	117	50	242	45	282
40	Jaunpiebalga Municipality	5	117.40	102.15	59	52	212	49	289
41	Jaunpils Municipality	N/A	N/A	N/A	N/A	N/A	N/A	N/A	N/A
42	Jelgava	14	51.43	22.24	51	41	61	11	103
43	Jelgava Municipality	8	92.88	45.41	87	55	134	25	157
44	Jekabpils	14	122.79	78.17	123	56	189	34	287
45	Jekabpils Municipality	4	187.75	103.29	177	99	288	98	300
46	Jurmala	18	33.39	13.47	32	23	44	13	58
47	Kandava Municipality	N/A	N/A	N/A	N/A	N/A	N/A	N/A	N/A
48	Karsava Municipality	N/A	N/A	N/A	N/A	N/A	N/A	N/A	N/A
49	Koceni Municipality	N/A	N/A	N/A	N/A	N/A	N/A	N/A	N/A
50	Koknese Municipality	N/A	N/A	N/A	N/A	N/A	N/A	N/A	N/A
51	Krimulda Municipality	5	86.40	43.95	88	44	128	41	146
52	Kraslava Municipality	4	223.50	67.97	219	160	291	151	305
53	Krustpils Municipality	4	80.75	39.79	96	39	108	23	109
54	Kuldiga Municipality	N/A	N/A	N/A	N/A	N/A	N/A	N/A	N/A
55	Kegums Municipality	5	33.00	3.39	33	30	36	29	38
56	Kekava Municipality	19	51.79	45.40	31	22	84	17	190
57	Lielvarde Municipality	9	43.33	26.56	37	21	65	12	93
58	Liepaja	28	55.29	44.57	42	23	84	10	227
59	Ligatne Municipality	4	64.50	58.02	45	24	125	19	149

60	Limbazi Municipality	14	89.36	79.09	57	37	139	23	298
61	Livani Municipality	4	55.50	3.70	55	52	59	52	60
62	Lubana Municipality	5	62.20	18.65	71	45	75	31	76
63	Ludza Municipality	7	46.57	47.55	38	9	57	8	144
64	Madona Municipality	5	109.80	32.11	98	89	140	83	162
65	Malpils Municipality	5	44.40	29.77	41	21	70	13	92
66	Marupe Municipality	2	5.50	0.71	6	5	-	5	6
67	Mazsalaca Municipality	8	94.63	71.60	73	33	172	23	206
68	Mersrags Municipality	5	97.20	74.21	104	26	165	13	199
69	Nauksenu Municipality	N/A	N/A	N/A	N/A	N/A	N/A	N/A	N/A
70	Nereta Municipality	8	129.13	75.07	116	87	143	52	299
71	Nica Municipality	4	48.25	11.95	48	37	60	34	63
72	Ogre Municipality	N/A	N/A	N/A	N/A	N/A	N/A	N/A	N/A
73	Olaine Municipality	16	17.13	15.85	13	7	23	4	70
74	Ozolnieki Municipality	N/A	N/A	N/A	N/A	N/A	N/A	N/A	N/A
75	Pargauja Municipality	4	70.00	9.02	70	62	79	59	81
76	Pavilosta Municipality	4	234.50	170.82	267	61	376	27	378
77	Plavinas Municipality	12	78.50	23.26	73	68	88	45	126
78	Preili Municipality	5	71.00	18.59	76	52	88	47	92
79	Priekule Municipality	4	250.50	163.71	225	111	416	106	446
80	Priekuli Municipality	4	77.00	34.47	80	44	107	32	116
81	Rauna Municipality	4	333.00	122.49	352	208	439	169	460
82	Rezekne	22	119.68	84.04	98	61	160	14	374
83	Rezekne Municipality	15	150.67	87.43	109	92	242	33	299
84	Riebini Municipality	N/A	N/A	N/A	N/A	N/A	N/A	N/A	N/A
85	Riga	144	32.74	31.86	25	14	41	2	279
86	Roja Municipality	10	76.60	57.35	52	30	123	15	171
87	Ropazi Municipality	N/A	N/A	N/A	N/A	N/A	N/A	N/A	N/A
88	Rucava Municipality	4	44.25	26.13	45	20	68	12	76
89	Rugaji Municipality	3	83.33	95.84	28	28	-	28	194
90	Rundale Municipality	5	41.40	15.34	48	26	54	22	59
91	Rujiena Municipality	6	87.33	58.08	81	38	129	27	182
92	Salacgriva Municipality	5	40.00	25.43	30	26	60	24	85
93	Sala Municipality	8	74.25	33.36	75	43	98	33	130
94	Salaspils Municipality	9	39.44	19.11	33	22	56	18	72
95	Saldus Municipality	4	22.50	11.03	21	13	34	13	36
96	Saulkrasti Municipality	4	35.50	1.73	35	34	37	34	38
97	Seja Municipality	N/A	N/A	N/A	N/A	N/A	N/A	N/A	N/A
98	Sigulda Municipality	8	94.50	33.45	98	62	121	52	147
99	Skriveri Municipality	7	97.29	69.02	67	43	146	33	224
100	Skrunda Municipality	4	107.50	88.23	92	34	197	32	214
101	Smiltene Municipality	N/A	N/A	N/A	N/A	N/A	N/A	N/A	N/A
102	Stopini Municipality	11	40.91	22.58	32	21	63	17	82
103	Strenci Municipality	9	34.44	22.61	29	13	56	11	74
104	Talsi Municipality	16	55.00	50.87	42	24	63	1	199
105	Tervete Municipality	N/A	N/A	N/A	N/A	N/A	N/A	N/A	N/A
106	Tukums Municipality	13	68.08	47.46	58	37	95	18	199
107	Vainode Municipality	4	51.75	9.03	53	43	60	40	61
108	Valka Municipality	N/A	N/A	N/A	N/A	N/A	N/A	N/A	N/A
109	Valmiera	53	42.74	29.92	33	23	56	6	122
110	Varaklani Municipality	8	148.38	80.27	113	95	234	52	273
111	Varkava Municipality	3	18.33	7.02	19	11	-	11	25
112	Vecpiebalga Municipality	5	83.00	60.81	79	35	134	23	182
113	Vecumnieki Municipality	5	133.80	59.49	141	83	182	46	210
114	Ventspils	15	50.63	25.20	29	20	42	6	102
115	Ventspils Municipality	8	50.63	29.04	62	18	76	11	81
116	Viesite Municipality	8	97.63	50.42	74	57	153	56	180
117	Vilaka Municipality	4	195.25	171.13	145	65	376	58	433
118	Vilani Municipality	4	139.50	83.32	129	66	224	61	240
119	Zilupe Municipality	8	43.38	23.27	39	29	62	10	85

n—number of measurements; N/A—data not available.
